# α-Synuclein at the Presynaptic Axon Terminal as a Double-Edged Sword

**DOI:** 10.3390/biom12040507

**Published:** 2022-03-27

**Authors:** Li Yang Tan, Kwan Hou Tang, Lynette Yu You Lim, Jia Xin Ong, Hyokeun Park, Sangyong Jung

**Affiliations:** 1 Institute of Molecular and Cell Biology (IMCB), Agency for Science, Technology and Research (A*STAR), Singapore 138667, Singapore; e0030861@u.nus.edu (L.Y.T.); e0426092@u.nus.edu (K.H.T.); e0324605@u.nus.edu (L.Y.Y.L.); e0408905@u.nus.edu (J.X.O.); 2 Department of Psychological Medicine, Yong Loo Lin School of Medicine, National University of Singapore, Singapore 119228, Singapore; 3 Faculty of Science, National University of Singapore, Singapore 119228, Singapore; 4 Yong Loo Lin School of Medicine, National University of Singapore, Singapore 119228, Singapore; 5 Division of Life Science, The Hong Kong University of Science and Technology, Clear Water Bay, Kowloon, Hong Kong; 6 Department of Physics, The Hong Kong University of Science and Technology, Clear Water Bay, Kowloon, Hong Kong; 7 State Key Laboratory of Molecular Neuroscience, The Hong Kong University of Science and Technology, Clear Water Bay, Kowloon, Hong Kong; 8 Department of Physiology, Yong Loo Lin School of Medicine, National University of Singapore, Singapore 117593, Singapore

**Keywords:** α-synuclein, Parkinson’s disease, Lewy bodies (LB), dementia with Lewy bodies, synucleinopathy, presynaptic axon terminal, α-synuclein oligomerization, synaptic vesicle endocytosis, targeted therapy

## Abstract

α-synuclein (α-syn) is a presynaptic, lipid-binding protein strongly associated with the neuropathology observed in Parkinson’s disease (PD), dementia with Lewy bodies (DLB), and Alzheimer’s Disease (AD). In normal physiology, α-syn plays a pivotal role in facilitating endocytosis and exocytosis. Interestingly, mutations and modifications of precise α-syn domains interfere with α-syn oligomerization and nucleation that negatively affect presynaptic vesicular dynamics, protein expressions, and mitochondrial profiles. Furthermore, the integration of the α-syn oligomers into the presynaptic membrane results in pore formations, ion influx, and excitotoxicity. Targeted therapies against specific domains of α-syn, including the use of small organic molecules, monoclonal antibodies, and synthetic peptides, are being screened and developed. However, the prospect of an effective α-syn targeted therapy is still plagued by low permeability across the blood–brain barrier (BBB), and poor entry into the presynaptic axon terminals. The present review proposes a modification of current strategies, which includes the use of novel encapsulation technology, such as lipid nanoparticles, to bypass the BBB and deliver such agents into the brain.

## 1. Introduction

Presynaptic defects are often observed prior to the onset of synucleinopathies and its associated neurodegenerative conditions, such as Parkinson’s disease (PD), dementia with Lewy bodies (DLB), and Alzheimer’s Disease (AD). Lewy body (LB) pathology, characterized by the excessive aggregation of oligomeric and fibrillar α-synuclein (α-syn), is one significant hallmark found in dopaminergic cells of the substantia nigra pars compacta (SNc) during the later stages of PD. LB is first observed in the olfactory tract, then the substantia nigra pars compacta, and later spreads to other parts of the brain. The dopaminergic neurons of the substantia nigra projects to the striatum of the basal ganglia, in which the degeneration of the striatal dopaminergic neuron explains the motor deficits and dyskinesia as with patients with PD.

Certain mutations and specific post-translational modifications, frequently those in the amphipathic domain, favorably facilitate α-syn nucleation, while disruption to monomer-to-oligomer ratios by the locus multiplication of α-syn gene (*SNCA*) is also associated with both PD and DLB [1,2]. However, recent studies have suggested that not all duplication and triplication events are penetrant, with some forms having different phenotypic outcomes [3]. Such evidence has suggested that different mechanisms may provide compensatory effects to maintain the highly conserved vesicle release at the presynaptic axon terminal.

α-syn was first isolated from the cholinergic synaptic vesicles of the torpedo ray and was thought to only localize at the presynaptic nerve terminals for synaptic transmission [4], especially in regions of the brain, such as the amygdala, hippocampus, and cerebral cortex [5]. Its specific localization to the presynaptic axon terminal is widely linked to its functions in presynaptic plasticity, neurodevelopment, cell viability, and the regulation of monoamines, especially dopamine [6]. In the presynaptic axon terminals, α-syn behaves as a soluble cytosolic protein, with about 80% of the protein reportedly bound to vesicles [7]. Interestingly, α-syn maintains a disordered, monomeric structure when exogenously introduced [8]. In dopaminergic neurons, the simultaneous genetic deletion of both α-syn and β-syn leads to selective changes in complexins and 14-3-3 protein expressions and is associated with a decline in dopamine release [9]. Furthermore, the presence of E46K α-syn interfered with the normal functioning of synapses, such as having a lower level of soluble NSF attachment protein receptor (SNARE) complexes that are involved in neurotransmission [10]. Given α-syn’s highly disordered features with hundreds of known and unelucidated conformations and interaction partners, the extensive repertoire of α-syn functions and proteoform presents as a worthy challenge to be fully characterized in the presynaptic axon terminal.

α-syn is a small pleiotropic molecule that can perform many critical functions in the presynaptic axon terminal. A-syn maintains proper neurotransmission and sustains cell survival [11], but early dynamic switches to more pathological conformations can trigger the onset of neurodegenerative diseases. We therefore liken the presence of α-syn at the presynaptic axon terminal to a double-edged sword that not only facilitates the survival of neurons but can also lead to their demise. In this review, we dissect the roles and functions of α-syn splice variants, domains and proteoforms within the presynaptic axon terminals. We highlight the potential mechanisms of oligomeric and fibrillar α-syn in causing neurodegeneration. In doing so, the current avenues for targeting mutant α-syn to modulate aberrant presynaptic defect in the early stages of PD/DLB could be referenced for future studies.

## 2. The α-Syn Protein

In total, there are three primary domains of α-syn and they have been well-documented. The amphipathic domain spanning residues 1–60 (Exons 2–4) at the N-terminus of α-syn contains six-to-nine spatially segregated, imperfect KTKEGV repeat sequences that extend into the central non-amyloid-β component (NAC) domain (residues 61–95) [12]. The primary role of the amphipathic domain is to anchor α-syn to anionic, small unilamellar vesicles (SUVs) or micelles through gangliosides and cholesterol as possible interaction partners [13]. Upon electrostatic interactions with lipid membranes [14], approximately two-thirds of α-syn folds into α-helical structures associated with its normal physiological functions. Mutant subtypes further crosslink to form the β–pleated strands associated with amyloid pathology [15].

Residues 61–95 (of the Exon 4) consist of the non-amyloid-β component (NAC) domain, which contains around two KTKEGV repeats that extend from the N-terminus. The NAC domain first garnered significant interest as a potential precursor of Alzheimer’s disease (AD) due to its association with aggregative activities [16], prior to the later revelation of the role of α-syn aggregation in PD. Interestingly, the NAC domain may also be involved in the transport of α-syn along the axon. Deletion of residues 71–82 prevented α-syn accumulations and blockages along axons, but also reduced α-syn’s localization to the presynaptic axon terminal [17]. This finding suggests that the residues 71–82 might be associated with microtubule-related motor or motor-associated proteins. Furthermore, the NAC domain’s capability to prompt synaptic clustering may also indicate a plausible role in the regulation of reserve pools analogous or complementary to the function of synapsins at presynaptic axon terminals [18].

The repeats at the far end of N-terminus (residues 6–25) anchor α-syn to the lipid membrane non-specifically, and with the highest affinity. Studies have shown that acetylation of the first 20 amino acids at the N-terminus promotes a partially formed α-helical state, enhanced binding to SUV membranes, and subsequently promotes an “initiation-elongation” process that induced α-helical folding towards the NAC domain [19,20]. In contrast, the central domain (residues 26–97) alone binds with variable affinities to membranes, depending on their specific lipid compositions [21]. Therefore, the central domain is more likely to act as the sensor of the lipid properties and regulator of binding affinity [22]. Intriguingly, the selective deletion of residues 41–50, 51–60, or 46–53 of the amphipathic domain did not seem to affect tetrameric or multimeric compositions in neuron-like cells. Neither does the missense mutations introduced within six to seven of the KTKEGV repeats promote tetramerization. The mutant monomeric forms are instead found to be neurotoxic, suggesting that tetrameric forms are more neuroprotective [23]. Put together, these findings highlight that the N-terminus end (residues 6–25) has a more functionally important role in modulating the multimerization of α-syn and binding of the protein to lipid membranes.

Finally, the C-terminus consisting of residues 96–140 and encoded by Exons 5–6 is a negatively charged, highly acidic (33% acidic residues), and proline-rich domain, which, in its native form, only weakly interacts with lipid membranes [21]. This domain is known to act as a chaperone for metabolic proteins during heat stress [24], and notably interacts with cationic targets, such as calcium ions [12,25]. A nuclear magnetic resonance (NMR) study showed that the binding of around 6–8 calcium ions to the C-terminus greatly enhanced the C-terminal membrane-binding affinity to other isolated vesicles. Consistently, calcium-induced binding of different vesicles to both the N-terminal and C-terminal ends were observed to lead to profound vesicular clustering at presynaptic axon terminals [12].

### 2.1. α-Syn Splice Variants and Isoforms

The splicing of certain exons may alter the properties of α-syn in a domain-specific manner. Canonically, alternative splicing of α-syn leads to four transcripts-the full length α-syn (α-syn-140) and three other truncated variants that lack either Exon 3 (α-syn-126), Exon 5 (α-syn-112), or both Exons 3 and 5 (α-syn-98). In normal physiological conditions, the full length α-syn-140 is expressed as monomeric forms in the presynaptic axon terminals within the substantia nigra, cerebellum, and prefrontal cortex [26]. On the other hand, the shorter α-syn-126 isoform is normally expressed in the substantia nigra and cerebellum [26].

Similar to α-syn-140, α-syn-112 is also endogenously expressed in the substantia nigra, frontal cortex, and cerebellum. The loss of Exon 5 is reported to have significantly enhanced α-syn’s phospholipid binding efficacy leading to impairment in synaptic vesicle recycling. When overexpressed, α-syn-112 oligomerizes rapidly dimerizes and trimerizes in vitro, at a much faster rate compared to α-syn-140, and interferes with clathrin-mediated synaptic vesicle endocytosis at the presynaptic axon terminal [27].

The excision of both Exons 3 and 5 leads to the shorter α-syn-98. Notably, this isoform has a loss of some segments of the amphipathic domain, but the NAC domain within Exon 4 is preserved. The injection of α-syn-98 into the mouse substantia nigra (SN) caused a significant increase in α-syn phosphorylation and rapid intracellular oligomerization. These outcomes led to neuronal atrophy and motor dysfunction, which are reminiscent of PD pathology [28]. Therefore, the combined findings from the splice variants posit that the loss of Exon 5 may have a more significant effect than Exon 3 in promoting α-syn oligomerization.

There is only a single account of the fifth less-known isoform, α-syn-41, from the substantia nigra of PD autopsy tissues [29]. This isoform is synthesized through the simultaneous excision of both Exons 3 and 4 to generate a prematurely truncated amino acid peptide at the N-terminal end. However, instead of the expected 78 amino acids protein, the eventual outcome is a 41 amino acid peptide because of a frameshift in the open reading frame. Therefore, α-syn-41 does not contain an NAC domain and the calcium-sensing C-terminus and is less likely transported along the axons due to a lack of residues 71–82. However, unlike the canonical isoforms that are localized within the cytosol, α-syn-41 is found mostly secreted out of the cell when overexpressed. The oligomerization of the α-syn-41 promotes its integration into the membrane, likely acting as a transporter channel that facilitates the reuptake of dopamine. Intriguingly, there is no apparent effect of the α-syn-41 overexpression on cellular viability [29]. The aggregation of α-syn-41 seems to be independent from the pathogenic activity of the other α-syn splice variants in vitro, but its detailed role in PD remains to be discovered.

### 2.2. Dynamic Alterations to 3D Structures of α-Syn-140

Most existing structural studies on α-syn focus on the full length α-syn-140 variant. Interestingly, the native structure of α-syn-140 is strikingly different from the structure of a typical globular protein. Instead of folding into a hydrophobic core, α-syn-140 presents natively as a mixture of rapidly equilibrating monomeric conformers, which, on average, contain little secondary structures made up of random coils and few β-sheets [30]. This unusual property of α-syn may be attributed to the concentration of hydrophobic residues in the central (NAC) region of the sequence and/or the role of the anionic C-terminal domain in preventing the aggregation. Intriguingly, this native disordered state is also purported to be stable for hours [31]. Upon the interaction with cell surface glycosphingolipid or phosphoinositol polyphosphate on the plasma membrane [32], the acetylated and partially coiled N-terminus region of α-syn rapidly penetrates the membrane. Within the membrane, this interaction induces the antiparallel helical structure to a change from a random-coil configuration to a more ordered helical state [33] displacing neighboring lipids and incorporating other monomers to the complex. Bound α-syn can exhibit a continuum of α-helical conformations, though its binding can be influenced by the charge of the lipid content on the membrane surface [34]. Some of the conformations favor the formation of pore-like structures that perforate the lipid bilayer [35]. Alternatively, α-syn may also aggregate via the barrel model, which consists of two helically coiled α-syn cross-linking and subsequently organizing itself into a β-pleated pore structures within the membrane [36,37]. In the latter model, barrel stave pores were formed with the progressive insertion of four helical units [37]. The barrel model is often associated with more pathological aggregates associated with synucleinopathies.

Apart from membrane interactions, α-syn-140 may also adopt conformations that resemble other proteins, such as tubulin or actin binders, calcium-calmodulin binders, and phosphatase or kinase inhibitors [38]. Likewise, α-syn has been associated with microtubule stabilizing protein Tau at the presynaptic axon terminal [39], and also has been found to be localized in the nucleus of the cell [40]. These findings suggest that different conformations of α-syn-140 have varied functions within and out of the synapses.

Interestingly, a truncation study also showed that residues 36–55 alone can orchestrate α-syn nucleation. When exogenously expressed, residues 36–55 adopt a β-hairpin structure, which readily assembles into toxic trimers in a hierarchical fashion. Sequentially, three copies of the trimer further assemble to form basket-shaped nonamers before forming an octadecamer. Molecular dynamic simulations suggest that the full length α-syn-140 may also form trimers through the same mechanism. These octadecamer complexes are able to interact with anionic lipid bilayer membranes and elicit cytotoxic effects in a similar fashion as α-syn-140, which suggest that 36–55 may be the most important site promoting nucleation [41].

α-syn may also undergo autoproteolytic (self-cleaved after translation) or proteolytic (cleaved by proteases) reactions to generate a diversity of thermodynamically stable products with unique and varied alterations to presynaptic functions. In addition to the acetylation at the N-terminus, other posttranslational modifications, such as phosphorylation, ubiquitination, nitration, oxidation, and dopamine-dependent adduct formation, also influence α-syn to form more toxic conformations. The overexpression of certain splice variants/proteoforms have been reportedly associated with PD and DLB pathologies. Unfortunately, the structures and functions of α-syn after these modifications have yet to be thoroughly examined. Much remains to be seen from the highly anticipated human proteoform project [42].

### 2.3. Involvement in Synaptic Vesicle Exocytosis

The direct role of α-syn in synaptic vesicle exocytosis has been extensively studied. In the presynaptic axon terminals, α-syn is involved in tethering vesicles at the reserve pool [43], transport of vesicles for exocytosis in a calcium-dependent manner [44], and the fusion of synaptic vesicle to the cell membrane [45,46]. α-syn binds to motor-associated proteins that facilitate vesicular transport and movement in the presynaptic axon terminals. A prime example is the Rabs family of motor-associated proteins. Rab1, Rab3A and Rab8A rescue dopaminergic neuron loss induced by α-syn [47]. α-syn facilitates vesicular localization to the membrane, possibly involved in formation of readily releasable pool (RRP) and interacts with SNARE complexes by binding to synaptobrevin-2/VAMP2 (V-SNARE) [48]. However, α-syn KO mice create contradictory outcomes, with some having reduced field excitatory postsynaptic potential (fEPSP) amplitudes in response to prolonged repetitive stimulation train at the hippocampal CA3-CA1 synapse, while other models showed no significant changes to electrophysiological properties [49]. Correspondingly, this may mean that other synucleins or synuclein-associated proteins may provide compensatory effects at the presynaptic terminals. Moreover, the roles of different α-syn isoforms and proteoforms requires further investigation and clarifications.

### 2.4. Involvement in Synaptic Recycling and Endocytosis

Apart from exocytosis, α-syn also plays an essential role in early endocytosis. In SH-SY5Y cells, α-syn facilitates clathrin-dependent endocytosis by interacting with phosphatidylinositol 4,5-bisphosphate (PI(4,5)P_2_) and clathrin adaptor protein 2 (AP2) at clathrin-coated pits [50]. The probability of α-syn binding to endosomes decreases downstream of the endocytic pathway, suggesting that α-syn is only involved in early endocytosis [51]. In the lamprey giant reticulospinal (RS) synapse, the introduction of excess α-syn dimers intracellularly led to a build-up of clathrin-coated pits (CCPs) with constricted necks that were still attached to the plasma membrane, a phenotype indicative of a vesicle fission defect [52], and also associated with dynamin-induced cleavage. Therefore, the ratio of polymers to monomers must be critically regulated in healthy synapses for maintaining adequate vesicular replenishment.

## 3. Pathological Impact of α-Syn Oligomers

### 3.1. Prevalence of α-Syn Mutations in Human Patients

The overexpression of α-syn, presumably by the hereditary amplification of the *SNCA* gene, is a leading hypothesis of synucleinopathies. However, epidemiological studies in PD/DLB patients showed a prevalence of only 1/2206 (0.045%) in the Swedish population, while triplication events account for 1.14% to 2.86% in African and Korean populations, respectively (Table 1). Together, these studies suggest that *SNCA* gene multiplication is a statistically rare event in clinical practice.

Mutations and post-translational modifications are known to induce α-syn nucleation. Point mutations within the *SNCA* gene were found in the amphipathic domain (A30P, E46K, H50Q, G51D, and A53T), and in the NAC domain (A76P). Phosphorylations are found in the NAC domain (S86), as well as the C-terminus (Y125, S129) in PD and DLB patients. The effect of these mutations in the pathogenesis of synucleinopathies have been well-established in many animal models. However, mutations in the SNCA gene are also uncommon, accounting up to only 4.5% of PD/DLB cases in the Greece population (Table 1). Despite low occurrences of these point mutations, studies performed using insertion/deletion (indel) or point mutations in animal models have their merits in elucidating the structural properties of individual functional domains of α-syn, which can offer interesting insights against specific α-syn domains for immunotherapies.

Intriguingly, other mutations contributing to familial PD and DLB are found within endosomal/lysosomal machinery related genes, such as leucine rich repeat kinase 2 (*LRRK2*) and *ATP13A2*, or in mitochondrial-related genes, such as protein deglycase *DJ-1*, PTEN-induced kinase 1 (*PINK1*), and *parkin* (Table 1). Together, these epidemiological insights suggest that there is not one single gene that is significantly associated with synucleinopathies, and that disruptions in mitochondrial respiration, the impaired ubiquitination/autophagy system may also influence α-syn’s capability to aggregate and contribute to PD/DLB pathology.

### 3.2. Effects of Mutations at the N-Terminus Domain of α-Syn

α-syn copy number gain is a rare event that affects only 0.045–4% of the world’s population (Table 1). About 75% of sporadic PD patients have low level copy number gains compared to control SNCA (45%) [74], which suggests that the effect of amplification is not fully penetrant. Similarly, amplification events have controversial outcomes in animal models. Many overexpression studies with constitutively-activated promoters showed altered synaptic transmissions and positive α-syn aggregation with altered behavior in animal models [75,76,77]. However, no Lewy body inclusions were observed in these studies, which suggest that overexpression in these models may not fully recapitulate the pathological progression of PD/DLB.

Missense mutations associated with PD/DLB within the N-terminus have variable outcomes, particularly with their binding affinities to plasma membranes. The A30P mutation at the N-terminus results in a lower membrane binding affinity, as the change in residue from an alanine to an inflexible proline removed a residue that can form a hydrogen bond, likely inhibiting helical coiling sterically [78], and redistributes α-syn away from synapses [79]. The N-terminal interaction is pivotal to promote α-syn tetrameric localization to SUVs and to prevent excess monomer build-up in the cytosol. Excess monomeric α-syn may interact with other proteins to promote neurotoxic effects. Otherwise, the C-terminal domain may also oligomerize upon calcium binding.

The E46K mutation is first reported as an autosomal dominant mutation in a Spanish family with familial parkinsonism and in three young asymptomatic subjects, but notably absent in healthy controls [57]. This missense mutation from a negatively charged residue (glutamic acid, E) to a positive-charged residue (lysine, K) resulted in the disruption of a salt bridge between E46 and K80, which typically diverts WT α-syn to a shallow energy minimum. The lack of electrostatic attraction therefore favors a different folding pathway that leads to the formation of a more stable, symmetrical double protofilament structure [80]. In stark contrast to A30P, this variant has significantly higher affinity to plasma membranes compared to WT α-syn. Mouse models with E46K exhibited motor impairment, and close histological examinations revealed α-syn inclusions that resembled Lewy body and Tau inclusions in neurons [81].

Although there is a switch from a non-polar residue (alanine) to a polar residue (threonine), the A53T variant possesses a similar binding affinity to plasma membranes as with WT α-syn. Theoretical models suggested that the A53T mutation interferes with the long-range interactions between the NAC region and the N-terminus or the C-terminus of α-syn [82], while NMR studies suggest that it stabilizes β-pleated sheet structures promoting fibril formation [83]. Cryogenic electron microscopy analysis showed that A53T α-syn fibrils are made up of two protofilaments twisted together into left-handed helices [84]. Correspondingly, A53T mouse models often did not show any observable signs of aggregation, such as inclusion bodies. However, AAV delivery of human A53T α-syn into mice still resulted in protracted neurodegeneration with significant nigral neuron loss by 17 weeks of age [85]. Electrophysiological analyses from the dopaminergic neurons of these mice, however, show profound changes to presynaptic activities, including an increase in presynaptic firing.

**Figure 1:**
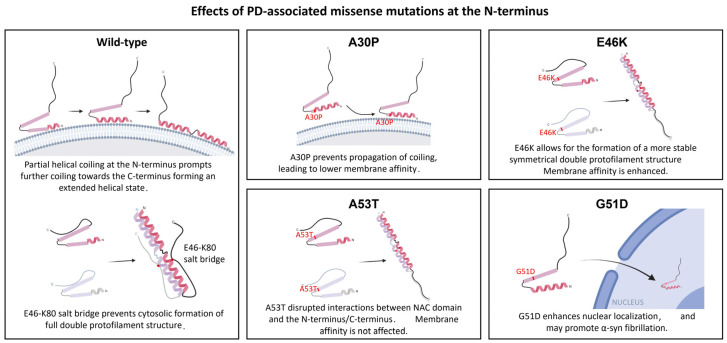
Effects of missense mutations on the α-syn structure and physiological functions. A30P may prevent further helical coiling of α-syn helical domain [32,33], reducing α-syn membrane-binding affinity [78,79], and allowing its aggregation within the cytosol E46K removes the salt bridge formation between E46 and K80, allowing the formation of stable protofilament that promotes further fibrillation [80]. Similarly, A53T interrupts distant NAC and N-terminal or C-terminal interactions, leading to protofilament formation [82]. G51D mutations promotes the nuclear localization and hyperphosphorylation of S129 that induces aggregation [59]. Figure is created with BioRender.com.

Remarkedly, a change of a small non-polar residue (glycine) to a large negatively charged aspartic acid at position 51 (G51D) has also been implicated in PD. Compared to those transfected with wild-type α-syn, primary neurons transfected with plasmid encoding for G51D mutant exhibit an enhanced level of colocalization of G51D mutant in the nuclear compartment. Furthermore, these neurons are reported to have higher levels of hyperphosphorylation at the S129 site of α-syn, which is known to be associated with the abnormal aggregation of α-syn [59]. Put together, this evidence determines that those different mutations and modifications of α-syn may bring about pleiotropic effects, all which eventually leads to the same phenotypic outcome of α-syn fibrillation (Figure 1).

### 3.3. Evidence of α-Syn Multimers Influencing Membrane Permeability

Different mutants of α-syn have variable affinities to plasma membranes. Variants of α-syn with sufficient membrane affinities (WT, E46K, and A53T) were reported to aggregate to form protofibrillar helical structures that act as voltage-dependent ion channels upon interaction with the plasma membranes containing anionic and curvature-inducing lipids. The depolarization potential required to open these channels are dependent on the type of mutations, with WT and A53T channels opening at around +100–150 mV, and E46K channels opening at around −50 mV transmembrane potential. Correspondingly, the A30P mutant with a lower affinity for the plasma membrane does not form similar pore-like structures [86]. Intracellular calcium ions appear to block the conductance of these channels. Excessive permeabilization at the membrane caused by these channels is likely to cause excitotoxicity leading to dopaminergic neuronal death.

A-syn is also a known transient interaction partner of dopamine transporters (DATs). Amphetamine-activated DAT-induced membrane depolarization enhances plasma membrane localization of α-syn, which in turn enhances DAT localization in cholesterol-rich membrane microdomains [87]. Intracellular aggregation of α-syn may prevent DAT localization to the plasma membrane, inhibiting dopamine reuptake into the synapses and reducing subsequent dopamine release.

### 3.4. Misfolded α-Syn Affects Normal Protein Recycling and Degradation

The ubiquitin–proteasome system (UPS) and autophagy are two major mechanisms known to degrade and recycle synaptic proteins at the presynaptic terminals. The UPS pathway is typically activated to target individual proteins, while autophagy degrades major cytoplasmic components and protein aggregates. In synucleinopathies, both pathways are found to be affected.

Ubiquitin is equally present within Lewy body inclusions of PD tissues. Tagging of ubiquitin to specific fated proteins, termed ubiquitination, serves to identify proteins fated for degradation by proteasomes. In cell cultures, the mammalian seven in absentia homologue-1 (Siah-1), an E3 ubiquitin-protein ligase, is reported to bind to E2 ubiquitin-conjugating enzyme UbcH8 to facilitate the mono- and di-ubiquitination of α-syn [88]. Interestingly, these post-translational modifications promote α-syn nucleation instead of proteasomal degradation. Similarly, autosomal recessive mutations on another E3 ubiquitin ligase, parkin, catalyzed the formation of a new 22-kDa glycosylated form of α-syn, which aggregates more rapidly [89]. These findings suggest a close link between disruption of normal UPS pathway in post-translational modifications that promotes α-syn nucleation and PD pathology.

Numerous lines of evidence have also shown that mutant α-syn aggregates can disrupt normal autophagic processes [90,91]. Extracellular oligomeric and fibrillar α-syn were uptaken by cells and found along endosome-to-lysosome and autophagosome-to-lysosome routes. In this model, the endocytic pathway, proteasome, and mitochondria functions were not affected. However, the study also identified that α-syn uptake decreased autophagosome clearance and dilated lysosomes, suggesting possible lysosomal defects upon extracellular uptake of fibrillary α-syn [38].

### 3.5. α-Syn Nuclear Localization May Alter Transcriptomic Profiles and May Be Neuroprotective

There have been multiple reports of α-syn being localized to the nuclear compartment, but its implication is unclear. A study showed that α-syn binds to retinoic acid before translocating to the nucleus in presence of calreticulin and Ca^2+^ [40]. In the nucleus, α-syn interact with histone proteins, crucial in maintaining a compact DNA structure [92]. At the nucleus level, α-syn forms a tight 2:1 complex with histones, and the fibrillation rate of α-syn is dramatically accelerated in the presence of histones in vitro. α-syn–histone complexes found in the nuclei of nigral neurons from mice exposed to a herbicide paraquat, which is structurally similar to 1-methyl-4-phenylpyridinium (MPP+) [92] could have a regulatory role by decreasing the pool of free histones available for DNA binding. Destabilized nucleosomes and transcription for inflammatory markers may enhance α-syn expression. This can expose genes to be expressed at a higher rate, which may lead to the enhanced aggregation of α-syn fibrils that eventually lead to neuronal death.

α-syn may play a neuroprotective role in the nucleus. Upon the genetic deletion of α-syn in mice, it has been reported that there is an increased level of DNA double-strand breaks (DSBs) and an impairment of repairing these DSBs in these mice cortical neurons, compared to wild-type mice with α-syn. Hence, the formation of α-syn fibrils in the cytoplasm may have reduced the compactness of histone–DNA complexes, making certain sections of the chromatin more susceptible to DNA damage [93]. Correspondingly, α-syn phosphorylated at the Serine-129 (S129) site was found at the site of DNA damage in the nucleus of mouse cortical cells, and associated with reduced nigral degeneration, while the non-phosphorylated form exacerbates nigral pathology [94].

Another possible role of α-syn includes directly binding to DNA binding protein that modulates DNA repair with implications for Lewy body disorders [93]. α-syn has been shown to colocalize with DNA damage response components within discrete foci in human cells and mouse brains. The removal of α-syn in human cells leads to increased DNA double-strand break (DSB) levels after bleomycin treatment and a reduced ability to repair these DSBs (can be rescued by transgenic introduction of alpha-synuclein). Serine-129-phosphorylated α-syn is rapidly recruited to DNA damage sites in the living mouse cortex. Cytoplasmic aggregation of α-syn reduces its nuclear levels, increases DSBs, and may contribute to programmed cell death via nuclear loss of function.

Mutations may also influence α-syn localization in the cells. The G51D mutation results in enhanced localization of α-syn in the nucleus, which suggests that the mutation may have affected its ability to be exported out of the nucleus or enhanced its import into the nucleus [59]. the A53T mutation increases α-syn localization to the nucleus and inhibits transcriptional adapter 2-alpha (TADA2a), which mediates histone acetylation [95].

Such epigenetic modifications and neuroprotective roles can also have an early profound impact on the transcriptomic profiles of nigral dopaminergic neurons. A study analyzing gene expression in the medial and lateral substantia nigra, as well as in the frontal cortex, found an overall global decrease in expression profiles, especially in genes that encode for subunits of mitochondrial-related proteins, such as respiratory complex I, and autophagy-related genes from the ubiquitin–proteasomal system (UPS) genes [96].

### 3.6. Fibrillar α-Syn Interacts with Cytoskeletal Structures

How α-syn affects the cytoskeletal network at the presynaptic terminal has been well-documented. Numerous studies have shown that α-syn aggregates affect the polymerization of actin filaments and microtubules through interactions with cytoskeleton-associated proteins, such as α-spectrin and Tau [97]. The microtubule-stabilizing protein Tau is another intrinsically disordered protein commonly associated with neurodegenerative disorders, such as Alzheimer’s disease (AD). Tau has been reported to interact with the C-terminal domain of α-syn, which leads to the formation of the toxic α-synuclein aggregates [39]. Tau hyperphosphorylation in AD influences the axonal transport and stability of microtubules along the axons and is therefore a putative cause of neurofibrillary tangles formation. Consistently, the α-syn A53T mutant affects Tau phosphorylation-dependent postsynaptic dysfunction in glutaminergic neurons, leading to impaired presynaptic structures and vesicular dynamics [98].

In the presynaptic axon terminal, the actin cytoskeleton plays pivotal roles in vesicular cargo transport, including endocytosis and exocytosis, the formation of the reserve pool of synaptic vesicles, and axonal growth and extension [99,100]. Presynaptic actin structures were profoundly affected in neurons with α-syn inclusions [97]. The A30P mutation in α-syn increases the rate of actin polymerization and disrupts the cytoskeleton during reassembly of actin filaments. Therefore, oligomerization from misfolding generally initiates an aggregation cascade that results in the formation of protein inclusion. Experiments with flies demonstrated that the disruption of the actin cytoskeleton induced by A53T α-syn was mediated by its interaction with α-spectrin, and caused the mislocalization of the mitochondrial fission protein GTPase dynamin-related protein 1 (DRP1) and subsequent mitochondrial dysfunction [101]. The latter study pointed to a direct relationship between α-syn and α-spectrin resulting in actin dysfunction that affects mitochondrial functions, which further have the detrimental effects of α-syn aggregation at the presynaptic terminals.

### 3.7. α-Syn and Mitochondrial Defects

Mitochondria with high membrane potential are preferentially found localized at presynaptic axon terminals. Studies suggest that these presynaptic mitochondria are the direct source of ATP for the assembly of actin cytoskeleton, which modulates synaptic vesicle and mitochondria clustering [102]. Indeed, α-syn overexpression and aggregation have been linked to mitochondrial dysregulation. The overexpression of α-syn in *Caenorhabditis elegans* resulted in increased mitochondrial fragmentation with altered morphologies [103]. The inhibition of α-syn expression restores fusion rates and returns mitochondria back to their tubular form. Interestingly, co-expressions of PD-associated genes PTEN-induced kinase 1 (*PINK1*), *Parkin*, and *DJ-1* prevented the fission of mitochondria caused by the overexpression of α-syn and restored mitochondria to their normal morphologies [103]. These findings suggest a close pathological relationship that tethers mitochondrial fusion–fission dynamics to α-syn.

*PINK1* and *parkin* are two autophagy-related mitochondrial genes that also function to maintain the integrity of the mitochondria [104]. Mutations in these genes were also found to cause abnormal swelling and reduced mitochondrial fission rates [105]. Similarly, a significant reduction in the colocalization of cytosolic GTPase dynamin-related protein 1 (DRP1) to the mitochondria also results in swollen mitochondria [101]. DRP1 is known to be crucial in regulating mitochondrial fission through translocation to the mitochondria from the cytoplasm [106].

The widespread use of pharmacological agents and toxins in PD models further provides proof for the intricate relationship between synucleinopathies and mitochondria dysfunction. Applications of respiratory complex I inhibitors rotenone and 1-methyl-4-phenyl-1,2,3,6-tetrahydropyridine (MPTP), a chemical precursor compound that is converted to the active toxin 1-methyl-4-phenylpyridinium (MPP+), are key examples of how defects in oxidative respiration may lead dopaminergic neurons to premature death [107]. The use of these complex I inhibitors induced significant oxidative stress, which resulted in dopaminergic neuron ablation at the substantia nigra pars compacta, and the eventual development of Parkinsonism-like symptoms [107]. The reasons for why and how dopaminergic neurons of the SNc are particularly susceptible to oxidative stress is still a pertinent question. With the current prevailing information, it is therefore likely that inherited mitochondrial defects may predispose the cell to early α-syn aggregation. In turn, the oligomerized form of α-syn further affects actin polymerization, causing dysregulated mitochondrial fusion–fission dynamics, reduced oxidative respiration, and the impaired generation of ATP at the presynaptic levels.

### 3.8. Mode of Oligomeric α-Syn Entry into Neurons

It has been shown that most extracellular α-syn can be internalized by neurons through endocytosis. A study with dopaminergic neuron-like cells has suggested that α-syn binds to DAT at the extracellular surface and exploited the DAT endocytic process to enter the cells [108]. Another study proposed that heparan sulfate proteoglycans (HSPGs) and solute carrier family 35 member B2 (SLC35B2) may also be extracellular receptors for endocytosis [109]. After internalization, the vesicles are transported to merge with autophagosomes, which subsequently accumulates fibrillar and non-fibrillar α-syn aggregates, before further merging with lysosomes to form phagolysosomes for degradation. 

The entry of α-syn fibrils by purely dynamin-dependent endocytosis have been proposed. Dynamin-1 is a GTP-binding protein purported to facilitate tubulin polymerization to form microtubule bundles in the process of receptor-mediated endocytosis [109,110]. Both pharmacological inhibitions using Dyngo (Dyngo 4A) and the loss-of-function K44A mutation to dynamin-1 prevents α-syn aggregation in early endosome vesicles [110,111]. In contrast, the application of Pitstop, a specific drug that inhibits the clathrin-mediated endocytic mechanism, led to no observable aggregation of α-syn [111]. The endocytic internalization of α-syn also involves the generation of sufficient force through the actin-related transport, via the activity of motor protein Myosin 7B in HEK293T cells [109]. 

Other modes of entry are also plausible. Knockout of parkin, a PD-associated E3 ubiquitin ligase, is also shown to accumulate caveolin at lipid rafts and promote uptake of α-syn through lipid rafts-dependent endocytosis [112]. As previously mentioned, extracellular α-syn may also directly interact with anionic charges of curvature associated proteins at cell membranes. This direct interaction causes α-syn to fold into a multimeric alpha-helical or beta-sheet structure that allows it to function as a transmembrane ion channel [113].

Signaling pathways may also facilitate oligomeric α-syn uptake. α-syn was reported to interact with cofilin-1, an F-actin depolymerizing protein, in promoting excessive actin polymerization which disrupts normal presynaptic actin architecture. Downregulation of cofilin-1 decreased α-syn aggregates entry, while simultaneous ROCK1 silencing and the pharmacological inhibition of Rho increased aggregate entry, suggesting that the Rho-ROCK1-LIMK-Cofilin-1 pathway can trigger aggregates entry into the host cells [114].

### 3.9. Mechanisms of α-Syn Secretion from the Presynaptic Axon Terminals

In healthy and pathological settings, both monomeric and multimeric forms of α-syn are detected in the blood plasma and cerebrospinal fluid [115]. An in vitro study overexpressing human α-syn in differentiated SH-SY5Y cells revealed that a significant amount of α-syn was detected in the culture medium as early as two hours and had accumulated over time [44]. The extent of multimerization of α-syn at both the intracellular and the extracellular environment is also likely associated with its expression within the cells. In cells expressing low levels of α-syn, only the monomeric form of α-syn was detected in both cell extracts and the conditioned medium. Studies using H50Q and A53T mutant variants also showed a significantly enhanced secretion of α-syn into the surroundings [116].

Secretory vesicles are also known to be generated by the conventional endoplasmic reticulum–Golgi apparatus (ER–GA) axis pathway [117]. The release of the aggregates is also mediated by exocytosis rather than membrane damage [44,118]. In SH-SY5S cells, α-syn is also found localized at the lumenal surface of secretory vesicles and more favorably aggregates within the lumen, rather than within the cytosol, which suggests that the lumenal environment could be optimal for the misfolding of α-syn [44]. Nevertheless, the addition of Brefeldin A (BFA), a classical inhibitor of the ER–GA axis pathway, did not fully inhibit the release of α-syn [44]. These findings suggest that α-syn can also be synthesized and released through other non-canonical routes, such as from the free ribosomes in the cytosol, and may directly pass through the plasma membrane.

At presynaptic axon terminals, synaptic vesicles are exocytozed via the depolarization of the presynaptic membrane arising from an axonal action potential. Can α-syn-laden vesicles be released extracellularly through similar mechanisms as with synaptic vesicles? An ELISA test performed on conditioned media extracted from primary cortical neurons treated with tetrodotoxin (TTX), (2R)-amino-5-phosphonovaleric acid (APV), and 2,3-dioxo-6-nitro-7-sulfamoyl-benzo[f]quinoxaline (NBQX) to block glutaminergic transmission indicated that a significant 70% of extracellular α-syn are secreted through activity-dependent mechanisms [119]. Therefore, α-syn release by secretory vesicles is likely orchestrated by a mix of mostly activity-dependent and less activity-independent mechanisms. Put together, these findings suggest that the ER–GA axis-associated vesicular exocytosis is likely to be the dominant mechanism for α-syn secretion.

Other modes of transport could also facilitate the cell-to-cell transmission of α-syn. The transfer of aggregates through direct cell-to-cell contact can be established through nanotube formation [120]. It has also been shown that exosomes are associated with the transmission of α-syn between neurons, and defects with lysosomal activity enhance the release of α-syn within exosomes [121]. Specifically, A53T and E46K α-syn interact with microtubule-associated proteins 1A/1B light chain 3B (LC3B), causing microaggregation at the surfaces of late endosomes and promoted the release of aggregates into the exosomes [122]. However, the amount of α-syn secreted through exosomes is predicted to be less than that through direct exocytosis [121,123]. The involvement of lysosomes is also intriguing, as this suggests that accumulated α-syn may be directed into exosomes when UPS or autophagy processes are impaired.

## 4. α-Syn-Based Treatment Techniques

Conventional drugs, such as levodopa (L-DOPA), dopamine agonists, and monoamine oxidase B (MAO-B) inhibitors, are the most common orally ingested, non-invasive treatment for PD, as it serves to counteracts the deprivation of dopamine release. For severe PD patients, high-frequency deep brain stimulation (DBS) is regarded as the last resort when all other methods fail to show any significant impact to alleviate motor symptoms. Furthermore, such treatment can only temporarily alleviate motor symptoms, but not non-motor symptoms, attribute to the rapid development of resistance, and does not address synuclein fibrillation as the root cause of synucleinopathies [124]. A long-standing hypothesis is that α-syn nucleation may be prevented by inhibiting the aggregative domain with targeted therapy. However, targeted treatment against α-syn using biologics or small molecules remains largely experimental and is proven to be quite difficult to achieve. Apart from toxicity and efficacy, the ideal methodology must fulfil the stringent criteria to bypass the blood–brain barrier, target extracellular α-syn, and enter into cells to target intracellular α-syn to prevent fibrillation.

Immunotherapies remain the most direct targeting technique to inhibit α-syn nucleation. Monoclonal antibodies designed to target specific epitopes on α-syn domains may block the direct interactions of extracellular α-syn to cell membranes and inhibit monomers of α-syn from interacting with each other. Some examples include the monoclonal antibodies 1H7, 5C1, or 5D12, which are designed to target the C-terminus of α-syn (residues 118–126). They were all shown to effectively reduce α-syn aggregation in vitro, rescued the loss of dopaminergic fibers in the striatum, and improved motor and memory deficits in mouse models [125]. Nevertheless, the efficacy of immunotherapies is likely largely limited due to their inability to target intracellular α-syn. Moreover, the presence of extracellular enzymes, such as matrix metalloproteinases and calpain, may also cleave α-syn into more aggressive truncated isoforms that no longer contains the epitope site for antibody binding [125,126]. In contrast to immunotherapies, other biologics that may readily enter into cell and bind to intracellular α-syn are also more likely to be more efficacious. β-synuclein (β-syn), an isoform and a binding partner of α-syn, is proposed to inhibit α-syn lipid-induced aggregation through competitive binding to the surface of lipid vesicles. Fragmented β-syn peptides with domains that interact with α-syn can permeate cells and showed considerable efficacy in reducing fibrillation in A53T *Drosophila* models [127]. However, just as other close members of synaptic-associated proteins, such as Shank2/Shank3, tend to play compensatory roles to preserve proper transmission [128,129], it is unclear if introducing β-syn fragments is effective in the long run. Although β-syn was found not to aggregate under normal physiological conditions, evidence suggests that β-syn may also oligomerize into long unbranched fibrillary amyloids similar to α-syn fibrils at high concentrations, in high temperatures, or in acidic conditions [130,131]. Therefore, other peptides or small molecules that have less risk for fibrillary aggregation are better alternatives.

The search for permeable, small, organic molecular compounds has also yielded several promising candidates that may inhibit aberrant α-syn nucleation. Squalamine, a steroid–polyamine conjugate first extracted from the Shark family *Squalus* and found to have some degree of broad spectrum antimicrobial and anti-angiogenic activity [132], was recently found to be efficacious for preventing α-syn membrane-driven nucleation. The compound carries a net positive charge and shows a high affinity for anionic phospholipids, which allows electrostatic adhesion without significantly disrupting the integrity of lipid membrane [133]. To date, squalamine is proven to be safe, with low toxicity in Phase I and II clinical trials [134,135]. Trodusquemin, another small organic molecule compound extracted from dogfish sharks, acts in a similar manner as squalamine, but is able to penetrate the BBB and also directly binds to the surface of α-syn fibrils to prevent aggregation [136]. Other papers also reported the new small molecular agents that can inhibit α-syn misfolding. The experimental small molecule NPT200-11 inhibits α-syn aggregation, thereby reducing cortical α-syn pathology, alleviates neuroinflammation, normalizes dopamine transporter (DAT) expressions at the striatum, and improves motor function in animal models [137]. A screening study also identified single-ring phenolic compounds 576,755 and 582,032 that inhibit oligomerization in vitro [138]. However, many of these early studies lack high resolution structural data to elucidate the mechanistic interactions between the small molecules and α-syn. Moreover, it remains to be seen if there are any small molecules that effectively prevent nucleation in Phase II and III trials, and if not, further screening for more efficacious compound is highly warranted.

## 5. Limitations and Future Directions

As with all neurological-related conditions, the most challenging aspect of treating synucleopathies is the delivery of the therapeutic agent into neurons. The blood–brain barrier (BBB) alone poses a high initial level of difficulty, but the requirement to target α-syn intracellularly adds another layer of complexity. Small organic molecules that are able to inhibit α-syn is rare, but still reported. However, such candidate small molecules need to be highly hydrophobic to permeate BBB and cellular membranes. Charged molecules, such as squalamine, have poor permeability across the BBB [136], yet having an ionic charge promotes the inhibition of membrane-induced nucleation and can allow more effective binding to domains of α-syn that forms ionic bonds and salt bridges. This tradeoff between bioavailability and efficacy serves as a critical dilemma for the selection of suitable therapeutic agents against synucleinopathies.

Apart from mass screening for a suitable molecule, an alternative method to effectively inhibit α-syn nucleation is to design synthetic peptides that can specifically target the N-terminus or C-terminus of α-syn. Such peptides need to have a complementary binding sequence with a high binding affinity to the aggregative domain of α-syn, but does not play a similar role as α-syn, or its imbalance may be subjected to other forms of amyloid pathologies with unknown consequential effects. Unlike charged molecules, peptides are known to permeate through the BBB and enter the brain through a combination of receptor and transporter mechanisms.

Fortunately, other modes of delivery into the brain and cells can be used to circumvent the issue of poor delivery. The encapsulation of small molecules, synthetic peptides, or peptide mRNA through lipid nanoparticles (LNPs) may be effective therapeutics for the delivery. Liposomal encapsulation renders a hydrophobic exterior, which allows permeation through BBB and allows for the release of its encapsulated products into the cells. The widespread application of LNPs in vaccines has been shown to have low cellular toxicity and mild symptomatic side effects [139]. In the context that the α-syn is mostly localized to the presynaptic terminals, it is ideal that there is a targeted delivery to the presynaptic terminals. However, one critical limitation is that LNPs, in general, have poor binding specificity and may transfect a diverse range of non-neuronal cell types. Future studies should investigate the binding of such LNPs specifically to the presynaptic terminals, by altering charged lipid compositions on the LNP surface, or by developing customized peptide–lipid nanoparticle conjugates [140,141].

Lastly, even if a suitable therapeutic agent has been identified, it is undecided when the treatment should be applied to the patient. Presynaptic defects have been posited to have occurred before the emergence of clinical symptoms. It is therefore plausible that it is more efficacious to use such therapies as a form of preventive medicine in pre-symptomatic stages, especially in family members of familial PD patients identified with rare forms of autosomal-dominant α-syn variations and mutations [142,143]. Further studies are required to observe the epidemiological aspects in human clinical trials and predict a treatment

## 6. Discussion

**Figure 2:**
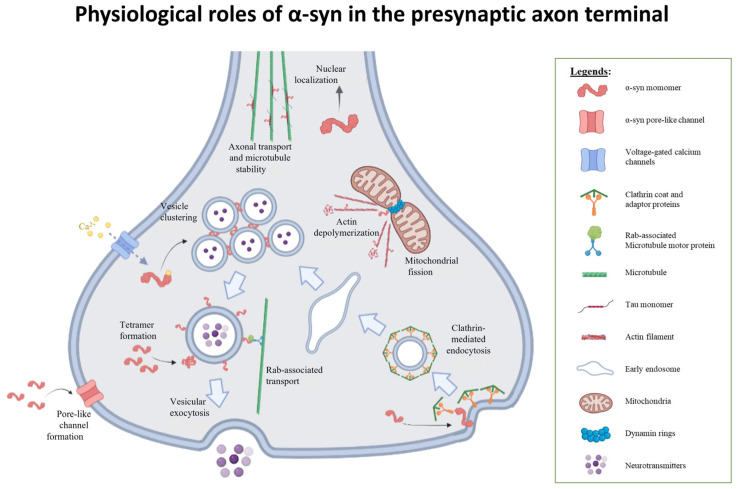
Physiological roles of α-syn in the presynaptic axon terminal. α-syn plays pivotal roles in the presynaptic axon terminal participating in vesicular clustering [12,18,43], recycling process, such as exocytosis and endocytosis [43,44,48,50,51,52], microtubule alterations [39], as well as maintaining mitochondrial fusion and fission dynamics through cytoskeletal reformation [99,100,101,114]. Figure is created with BioRender.com.

It is clear that the different isoforms and extent of α-syn polymerization have complex physiological roles at the presynaptic terminals. Firstly, monomeric α-syn finely regulates exocytosis in an activity-dependent manner. The N-terminus of α-syn attaches to synaptic vesicles, while the anionic C-terminus can interact with other proteins for trafficking. After calcium-ion binding, the C-terminus also adopts a helical structure and attaches to other vesicles, promoting vesicular clustering, which may be intricately linked to presynaptic plasticity. Secondly, α-syn may interact with clathrin coats to generate recycling vesicles via clathrin-mediated endocytosis. Thirdly, the polymerization of α-syn into tetramers can also be important for binding to v-SNAREs in the active zone (Figure 2). Put together, these findings suggest that other synucleins or synuclein-associated proteins may also have synergistic or compensatory roles at the presynaptic terminals.

**Figure 3:**
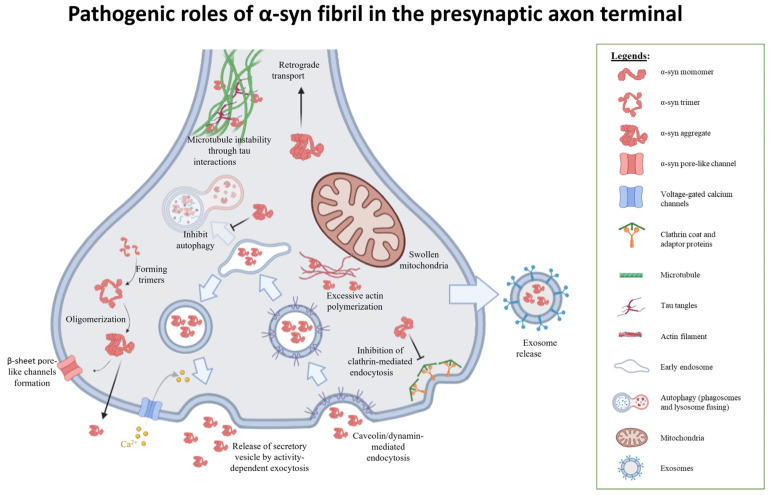
Putative effects of pathogenic α-syn in the presynaptic axon terminal. α-syn monomers forms trimers [27,41], which further oligomerizes to form amyloid fibrils [41], or β-sheet-like pores at the cell membrane [36,37]. Fibrils may interfere with cytoskeletal polymerization and depolymerization dynamics [97,98,101], mitochondrial fusion/fission dynamics [101,105,106], impairs clathrin-mediated endocytosis [27], and inhibiting autophagic systems [38,90,91], to cause a pleiotropic range of effects on the presynaptic axon terminal. Furthermore, α-syn fibrils may be released and taken in at the presynaptic axon terminal [110,111,119], suggesting the bidirectional transport of α-syn across neuronal synapses. Figure is created with BioRender.com.

Regardless, both intrinsic and extrinsic modifications to the α-syn monomer-to-polymer balance can act as a significant switch to induce the onset of synucleinopathies. Excessive monomeric α-syn, as opposed to its tetrameric form, creates a toxic environment that induces neuronal atrophy. The aberrant oligomerization of α-syn caused profound fibrillary aggregation at the presynaptic axon terminal, disrupted mitochondrial fission mechanisms, impaired autophagic process, and disrupted intracellular cytoskeletal structures, all of which can impair the release of neurotransmissions and enhance neuronal susceptibility to death. Furthermore, α-syn may be released through activity-dependent exocytosis, exosomes, or through nanotubes (Figure 3). Put together, these findings indicate that α-syn has a central, multifaceted role for modulating synaptic transmissions, but several risk factors may prompt α-syn aggregation, thereby seeding synucleinopathies.

A major form of synucleinopathy is PD, which is regarded as the second most common neurodegenerative disease worldwide [144]. The generic symptoms of PD are segregated into the four cardinal motor symptoms, such as resting tremor, dyskinesia, postural instability, and rigidity, as well as non-motor symptoms, such as autonomic dysfunction, gut dysbiosis, loss of sleep, and dementia. Extensive histological studies reveal two neuroanatomical locations, the olfactory nuclei and the medulla oblongata, as the earliest pathophysiological evidence of Lewy body (LB) formation in the brain. Through these findings, Braak and colleagues hypothesized that idiopathic PD most likely arises from the olfactory epithelium or the intestines [145,146], possibly arising from an altered gut microbiota population [147]. In this theory, α-syn is likely a paracrine signal that may be released by enteric endothelial cells as a proinflammatory response. Calcium-dependent calpain proteases are released by inflammatory cells, which cleaves α-syn into truncated isoforms that are more prone to aggregation [148].

Braak’s postulate is later supported by pathogenesis studies through α-syn injections into the gut of mouse models. In a recent study, fibrillary α-syn was observed to be taken up by enteric neurons at the gut and was transported along the axonal fibers of the vagus nerve from the gut to the midbrain [149]. Extracellular α-syn at synapses electrostatically interact with the extracellular presynaptic membrane, integrating into the lipid bilayer first as a transmembrane protein, before oligomerization into pore-like structures at the membrane [35]. Thus, in the normal physiological system, α-syn may be regarded as an important retrograde signaling molecule that induces neurotransmitter release in response to environmental stress. Since the vagus nerve comprises of a vast majority (80%) of afferent parasympathetic and sympathetic fibers, dysfunctional synaptic vesicle release due to the uptake of α-syn fibrils into the presynaptic axon terminals of these neurons are likely to manifest as vagal autonomic nervous system (ANS)-associated non-motor symptoms. This may explain the clinical presentation of gut dysbiosis, which precedes motor symptoms in early PD [150].

At the later stages of the disease, Lewy body (LB) pathologies infiltrated from the SNc to the striatum basal ganglia and propagated beyond to different cortical regions. The appearance of the LB pathologies in the interconnected pathways have some influence on the phenotypic outcome. The presence of LB in striatal dopaminergic neurons from the SNc may interfere with normal striatal regulation, which manifest as motor symptoms, such as dyskinesia and rigidity. Other symptoms may emerge when other cholinergic, serotonergic, noradrenergic, glutaminergic, and GABAergic pathways, such as the prefrontal cortex, are being affected [151]. Particularly, the dysregulation of the noradrenergic and serotonergic pathways may contribute to non-motor symptoms, such as depression [152,153]. Reduced cholinergic stimulation can contribute to motor-learning degradation and poorer adaptive motor responses [154].

Despite a clear association between α-syn build-up and PD, how the misfolding of α-syn is induced in the early stages of PD, remains unclear. Amplification and genetic mutations of the SNCA gene have been implicated in PD/DLB, but the prevalence rates are exceedingly low. Alterations to mitochondrial and UPS-associated genes were also reported (Table 1), indicating that there is no single gene or pathway that is significantly causal. As such, presynaptic mitochondrial-induced oxidative stress and autophagic defects are also likely to have promoted α-syn misfolding early in PD/DLB. Over time, misfolded α-syn is likely to nucleate with other proteins at the presynaptic axon terminals, before being transported to the soma, eventually forming Lewy bodies, becoming pathogenic, and therefore contributing to the subsequent development of synucleinopathy.

Other glial cells in the brain parenchymal are also influenced by the build-up of extracellular α-syn fibrils. Accumulations of excessive α-syn fibrils directly bind to microglial TLR2 receptors [155], by triggering a more reactive state that promotes phagocytic exhaustion and the excessive production of proinflammatory cytokine release [156,157,158,159], thereby releasing oxidative products and toxic kynurenines, a class of tryptophan metabolites [160]. These products lead to an excessively toxic environment and the selective recruitment of peripheral immune cells, which lead to the premature death of the striatal dopaminergic neurons at the SNc.

A key consideration in the treatment of PD is to alleviate the psychiatric or neurological implications, in which timely treatment can drastically improve the quality of life of PD patients. Depression, mental disorders, sensory complaints, sleep disturbances, and a loss of body presentation are characteristic symptoms of PD [161,162], but many of these traits are heterogeneous in PD patients. Depression is a prominent example, in which its prevalence averages at 35%, but ranges widely from 2.7% to 90% in different studies [163]. Fortunately, other associative tests may also be used as correlates for early diagnosis and/or prognosis. Recent magnetic resonance imaging (MRI) scans for depressed PD patients revealed distinguishable widespread alterations to numerous neuroanatomical regions, including the neocortex, cerebellum, brainstem, basal ganglia, thalamus, and limbic regions. Machine learning using multinomial tensor regression analysis (MTRA) of these brain MRI scans presented a high accuracy (94%) for differentiating between non-depressed PD, depressed PD patients, and healthy controls [164]. 

In some cases, PD patients also exhibit aberrant multisensory integration, which can be detected through other unconventional tests. One study showed that PD patients have declined visuospatial abilities, which is observed as an absence of fearful expressions against an invasion of peripersonal space [165]. This trait can be observed through visuotactile trials by the detection of conductance response on the facial skin upon visual stimulation of an approaching human face with varied emotions [166]. PD patients with reduced ventrolateral premotor activity also have a reduced ability to recognize facial emotions [167]. A study utilized single-pulse transcranial magnetic stimulation (TMS) to investigate corticospinal motor excitability for emotion perception [168]. Some PD patients also showed poorer tool embodiment outcomes, which are elucidated from executing pointing movement and tactile estimation tasks [169]. These findings suggest that different modes targeting different aspects of non-motor symptoms may be used as correlates for PD-associated neurocognitive alterations, which may be used for diagnosis or adopted for preclinical studies in animal models and clinical trials.

Nevertheless, to define the best treatment regimen for PD, several pertinent questions still persist. Can the inhibition of α-syn infiltration into the presynaptic axon terminals treat PD? Can the oligomeric and fibrillar α-syn be targeted to prevent pathogenic spread? How do the splice variants, mutations, and phosphorylation of α-syn influence presynaptic axon terminals? Put together, the recent findings reveal exciting avenues for further research into the field of synucleinopathy, but more work must be performed to fully elucidate the functionality of the α-syn at the presynaptic axon terminals. In turn, it is hopeful that results obtained from animal model studies or clinical trials of non-motor symptoms, such as visuotactile conductance tests on facial skin and corticospinal motor excitability, can be used to diagnose and quantify the efficacy of treatment for PD. These multimodal studies (e.g., conductance studies and MRI) may contribute to further insights into potential treatment plans during the developing stages of synucleinopathies, and hopefully provide a basis for the decision of treatment outcomes in PD clinical trial targeting early PD patients.

## 7. Conclusions

In its native forms, α-syn is a highly intrinsically disordered protein that interacts intracellularly with many molecular targets to facilitate synaptic vesicle exocytosis, endocytosis, and can also function as protein channels, and modulate mitochondrial respiration rates and nuclear transcriptions at the presynaptic axon terminal. However, unknown factors may prompt α-syn to misfold into a prion-like state, which promotes nucleation into fibrillary structures with altered physiological properties. In turn, this impairs neuronal synaptic transmission and survival. Increasing evidence suggests that these fibrillary aggregates can be released from the presynaptic axon terminals and taken up at the presynaptic axon terminals, suggesting a plausible bidirectional propagation of fibrillary α-syn cell-to-cell across synapses. Inhibiting α-syn domains and membrane interactions both intracellularly and extracellularly may effectively inhibit α-syn fibrillation and prevent toxicity in the early stages of the disease. Future interventions should continue to screen for suitable small organic molecule inhibitors with a high binding affinity to respective domains and should also have high BBB permeability. If this is not possible, packaging efficacious inhibitors within lipid nanoparticles may otherwise be performed to improve hydrophobicity and entry into the brain.

## Figures and Tables

**Table 1:** Prominent PD/DLB mutations and modifications and their associated prevalence. In general, PD/DLB associated mutations and modifications are rare within the *SCNA* gene, with most alterations being found in the N-terminus of the α-syn protein. On the contrary, mutations in other genes associated with the mitochondria, the ubiquitin–proteasome pathway, and cytoskeleton are more prominent.

Gene	Mutations/Modifications	Population	Prevalence in PD/DLB Population	Reference
*SNCA (PARK1*)	Gene duplication	Sweden	1/2206 (0.045%)	[53]
	Gene triplication	Korea	2/70 (2.86%)	[54]
		South Africa	1/88 (1.14%)	[55]
	A30P (N-terminus)	Germany	5/199 (2.51%)	[56]
	E46K (N-terminus)	Spain	10/337 (2.97%)	[57]
	H50Q (N-terminus)	British	1/451 (0.22%)	[58]
	G51D (N-terminus)	France	4/443 (0.90%)	[59]
	A53T (N-terminus)	Greece	5/111 (4.5%)	[60]
		Korea	1/72 (1.39%)	[61]
	Y125 phosphorylation (C-terminus)	British	3/4503 (0.067%)	[62]
*Parkin (PARK2*)	Copy number variations, small deletions/insertions	Northern Italy	164/3603 (4.55%)	[63]
	Point mutations/small deletions, exon rearrangement	Mixed	17/100 (17%)	[64]
*Omi/HtrA2 (PARK3*)	Point mutation	Germany	4/892 (0.45%)	[65]
*UCHL1* (*PARK5*)	Point mutation	Germany	4/507 (0.79%)	[66]
*PINK1 (PARK6*)	Point mutations (e.g., M129L, P153R)	Malaysia	6.9%	[67]
		Tunisia	2.5%	[68]
	Exon deletion	Iran	Familial: 6/130 (4.6%)	[69]
*DJ-1 (PARK7*)	Exon deletion	Iran	Familial: 3/130 (2.3%)	[69]
	Point mutation	Mixed	EOPD: 2/185 (1.1%)	[70]
	Exon deletion, splice site changes	Mixed	2/100 (2%)	[64]
*LRRK2 (PARK8*)	Point mutations	Sweden	0.54%	[53]
		Italy	Overall: 13/629 (2.1%) Familial: 9/177 (5.1%) Sporadic: 4/452 (0.9%)	[71]
		Italy, France	Italian (familial): 6/123 (4.8%) French (familial): 2/126 (1.6%)	[72]
*ATP13A2 (PARK9*)	Point mutations	Japan	28/117 (23.9%)	[73]
	Exon deletion, gene duplication, gene triplication	Iran	Familial: 10/130 (7.7%)	[69]
*GIGYF2 (PARK11*)	Point mutation	Italy, France	4.8%	[72]

## Data Availability

Not applicable.
